# Editorial: Oncolytic virotherapy

**DOI:** 10.3389/fmolb.2023.1287885

**Published:** 2023-11-01

**Authors:** Ahmed Majeed Al-Shammari, Pier Paolo Piccaluga

**Affiliations:** ^1^ Experimental Therapy Department, Iraqi Center for Cancer and Medical Genetic Research, Mustansiriyah University, Baghdad, Iraq; ^2^ Biobank of Research, IRCCS Azienda Opedaliera-Universitaria di Bologna, Bologna, Italy; ^3^ Department of Medical and Surgical Sciences, Bologna University School of Medicine, Bologna, Italy

**Keywords:** immuno-virotherapy, newcastle disease virus (NDV), combination therapies, hsv, T-VEC

## 1 Background and purpose of the Research Topic

**FIGURE 1:**
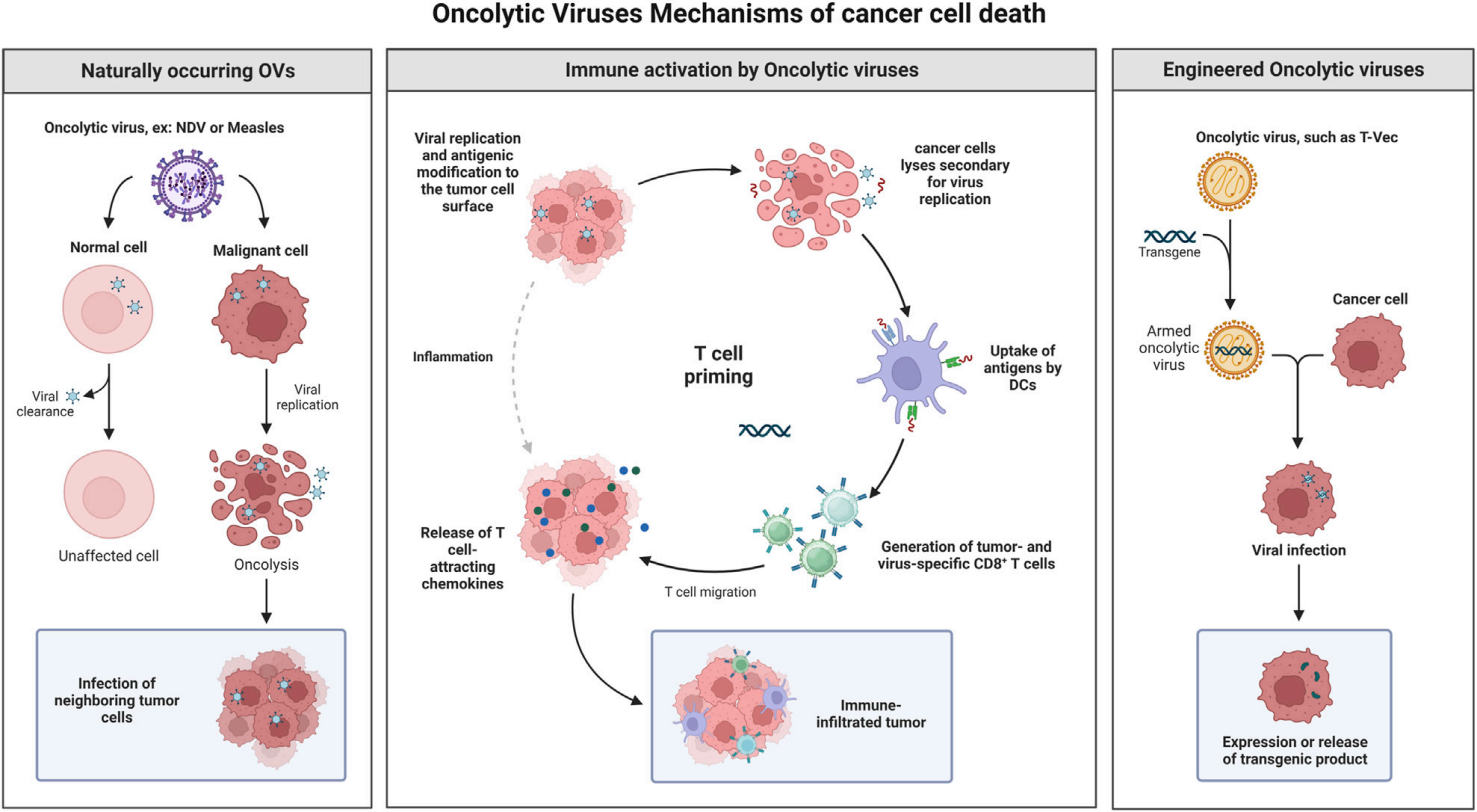
Oncolytic virotherapy is divided into two main groups, according to tumor specificity: naturally oncolytic viruses replicating in human cancer cells and gene-modified viruses engineered to accomplish selective oncolysis. OV kills cancer cells through several mechanisms, including cell lyses, due to virus replication, apoptosis induction, induction of specific antitumor immune response, and transgene expression that lead for cancer cell death and many others. Created with BioRender.com.

Using viruses to treat cancer is an established concept, and many viruses have shown promising antitumor efficacies (Vähä-Koskela et al., 2007). Oncolytic viruses are safe and well characterized pathogens with a stable genome (Maroun et al., 2017). The outstanding clinical results of oncolytic virotherapy deserve serious attention and consideration to make it a treatment option alongside classical cancer therapeutics (Russell et al., 2022). Virotherapy uses replication-competent oncolytic viruses to replicate and destroy cancer cells selectively. The transformed nature of cancer cells offers a permissive environment for the replication of some viruses and to complement viral mutations (Nemunaitis and Edelman, 2002). *In situ* amplification and spread within the tumor mass are the key benefits of such replication-competent viruses. Oncolytic virotherapy is divided into two main groups, according to tumor specificity: naturally oncolytic viruses to replicate in human cancer cells and gene-modified viruses engineered to accomplish selective oncolysis (Driever and Rabkin, 2001). OV kills cancer cells through several mechanisms (Figure 1), including cell lyses, due to virus replication (Al-Shammari et al., 2021).

In this Research Topic, 15 articles related to oncolytic virotherapy were submitted, including 8 reviews, one method article, and six original research articles. These articles shed light on recent and promising research on oncolytic virotherapy and ways to enhance its efficacy against cancer.

The articles were divided into five subtopics: 1) cancer models to investigate the efficacy of oncolytic viruses; 2) OVs as cancer immunotherapeutic agents; 3) novel viral platforms; 4) combination therapies; and 5) methods to develop OV quantification.

## 2 Cancer models investigate the efficacy of oncolytic viruses

Carter and colleagues (Carter et al.) used stable organoid cell cultures derived from breast cancer tissue to develop a protocol to study the effects of oncolytic viruses. They used an established three-dimensional organoid model derived from tissue from 10 patients with primary breast cancer.

They developed an investigational protocol for oncolytic viruses’ infection of organoid cultures. They compared the oncolytic effects of the measles vaccine virus (MeV) and a vaccinia virus (GLV) genetically engineered to express different transgenes. The most significant oncolytic effects were observed with oncolytic viruses expressing a suicide gene combined with the prodrug 5-FC. Therefore, the *in vitro* cancer model offers testing methods for new virotherapeutic vectors for treating breast cancer for further use *in vivo*.

Salman, Al-Shammari and colleagues (Salman et al.) established 3D coculture spheres *in vitro* consisting of two types of cells: the first type is derived from the breast cancer cell line (MCF-7 or AMJ13), and the second type is normal adipose tissue-derived mesenchymal stem cells. The floater culture plate was used to culture the cells so they could form spheroids, which were transferred to a special scaffold dish. The newly formed 3D culture spheroids were used to assess the oncolytic activity of the Newcastle disease virus AMHA1 strain by labeling the Newcastle disease virus (NDV) with a fluorescent PKH67 linker to track virus infection. They found that introducing chemical fluorescent dye into NDV particles is an effective strategy to identify virus particles in the infected co-culture spheroid model. Their results revealed that the oncolytic strain of NDV AMHA1 replicates effectively in cancer cells but not in normal cells in the 3D coculture system. This combined model of 3D coculture plus fluorescent tracking indicates that the NDV AMHA1 strain is selective and effective in antitumor virotherapy.

Howard and his associates (Howard et al.) evaluated the systemic delivery of HSV1716 in multiple mouse models of breast cancer. They found a direct relationship between virus tolerability and mouse strains. They tested the C57/B16, FVB, and Balb/c strains and found different responses. Intravenous administration of OV induces a lethal side effect in Balb/c mice, while C57/B16 is the most tolerant. These differences in response to OV in mouse strains may produce confusing results, which are mainly due to the interaction between OV and the immune system of these different strains. Eventually, this leads to a decrease in predictive value and low clinical efficacy. Howard and associates treated Balb/c mice with immunomodulators before injecting OV to decrease side effects. However, they could not estimate whether the immunomodulators increased virus tolerability. However, this work presented data to support therapeutic modulation of immune subsets with the aim of promoting a pro-inflammatory reaction, particularly by increasing CD8 + T cell levels that combat tumor growth. Finally, they are stating that the inconsistencies found in mouse models will help to have a larger picture, making it applicable, possibly to heterogenic human populations, which will lead to the development of translational oncolytic virotherapy.

## 3 Oncolytic viruses as cancer immunotherapeutic agents

Cerqueira and colleagues (Cerqueira et al.) reviewed possible immunotherapies that can be combined with oncolytic viruses for the treatment of Melanoma according to its molecular characteristics. They explored molecular changes in Melanoma that can be targeted taking into consideration that melanoma has a high mutation rate, leading to the appearance of tumor-specific antigens (TSA) and infiltration of lymphocytes, facilitating the utilization of therapeutic technologies that elicit novel or reinstate preexisting responses from the immune system. Immune checkpoint inhibitors are one of these innovative therapeutic technologies for melanoma treatment that could be combined with oncolytic virotherapy for synergistic action. They propose that a virus’s oncolytic action is due to virus replication and activation of innate and adaptive antiviral immune responses. This may be just as important, if not more so, than viral replication.

A research work by Uche et al. engineered recombinant oncolytic HSV-1 (oHSV) VC2-OVA that expresses a fragment of ovalbumin (OVA) as a fusion protein with the virion capsid protein VP26. They evaluated the efficacy of VC2-OVA to work as a vector capable of stimulating specific antitumor immunity in a syngeneic murine melanoma model. The administration of VC2-OVA through therapeutic vaccination resulted in a notable decrease in the presence of tumor cells in the lungs of mice that were intravenously exposed to B16cOVA cells. Furthermore, the administration of VC2-OVA resulted in strong preventive antitumor activity and prolonged the survival of mice that were intradermally implanted with B16cOVA tumors compared to mice inoculated with a control virus. Their findings demonstrate the efficacy of VC2 as an oncolytic virotherapy, showing promise for its potential application as a combined oncolytic virotherapy and personalized vaccine in the treatment of human and animal malignancies.

In the review by Kaufman et al., they described the progress made in advancing Talimogene laherparepvec (T-VEC) to the most proper melanoma patients, expansion to patients with non-melanoma cancers, and clinical trial results of T-VEC combination studies. T-VEC is a modified oncolytic herpes Simplex virus type 1 (HSV-1) that encodes granulocyte-macrophage colony stimulating factor (GM-CSF). T-VEC is adapted for selective replication in melanoma cells, and GM-CSF was expressed to augment host antitumor immunity. T-VEC is indicated for the local treatment of recurrent melanoma after primary surgery and is the first-in-class oncolytic virus to achieve FDA approval in 2015. Its tumor cell selective replication was improved by careful deletion of the two viral infected cell protein (ICP) 34.5 genes, which encodes the neurovirulence factor. Furthermore, deletion of ICP47 is believed to promote antitumor immunity as it facilitates MHCI loading of tumor-associated antigens. It showed very promising clinical outcomes. Additionally, it appeared that T-VEC could be used in combination with or sequentially to checkpoint blockade, without influence on therapeutic responses. T-VEC may be an important consideration for older patients with melanoma who may not be able to tolerate other systemic options.

According to the findings reported by Kaufman et al., there is evidence indicating the existence of specific patient subsets within the melanoma population who may exhibit a higher likelihood of seeing therapeutic benefits from T-VEC treatment. Mostly, patients with head and neck melanoma appeared to have higher response rates. Despite the higher mutation load due to Sun exposure being postulated, no demonstration of this was offered. Other potentially interesting settings are allotransplanted patients who cannot receive potent immunotherapy, such as immune checkpoint blockade, due to the risk of rejection of allograft, as well as patients with early-stage I-II melanoma patients, such as neoadjuvant treatment. As far as other cancers are concerned, T-VEC showed *ex vivo* preclinical activity, and then clinical trials have been planned. In general, accessible tumors for intratumoral injection have been a priority, and this has included head and neck squamous cell carcinoma, soft tissue sarcoma, and breast cancer. Other studies have been conducted to evaluate T-VEC in pancreatic cancer, hepatocellular carcinoma, and non-melanoma skin cancers. The combination with CTLA4 blockers seems promising, but further investigation is needed. It should be noted that some data support the role of elements of the antiviral interferon signaling machinery in tumor cells as possible predictive biomarkers of oncolytic virus (OV) activity and merit further clinical investigation (Kaufman et al.).

## 4 Novel viral platforms

A review article (Cristi et al.), described genetic modifications that were done to OVs to improve the killing ability of tumor cells directly, to dismantle the tumor microenvironment, or to alter tumor cell signaling and enhance antitumor immunity. Although many OVs have progressed to human clinical trials, their performance as monotherapy has not been as successful as expected. Importantly, recent literature suggests that the oncolytic potential of these viruses can be further increased by genetically modifying the viruses.

These advances are particularly important to increase virus spread and reduce metastasis, as demonstrated in animal models. The extracellular matrix (ECM) in a tumor does not have the same characteristics as that in normal tissues. In the tumor, the ECM is more rigid, abundant and dense. Because of this, tumor ECM acts as a barrier for therapeutic agents such as OV. At the same time, the barrier impairs oxygen and nutrients supply, activating apoptosis and senescence. Thus, ECM is a candidate cancer therapeutic target. Since at the onset of metastasis, during the invasion process, remodeling of the ECM is mainly done by metalloproteases (MMPs), both adenovirus- and vaccinia-based OVs have been genetically modified to exploit the natural functions of MMPs and enhance virus dissemination. Other attempts have been made by adding relaxin, hyaluronidase, or exonucleases. Overall, it resulted in increased virus spread and reduced tumor growth, including metastases in some cases (Cristi et al.).

Recently, Li and colleagues (Li et al.) showed that Ad-Apoptin-hTERTp-E1a (Ad-VT), a bispecific oncolytic adenovirus, can effectively induce cell death of breast cancer cells and has a better effect when used in combination with chemotherapy drugs (1–2). Ad-VT has no cytotoxicity in normal cells, with the advantage of specifically inducing tumor cell apoptosis, through the expression of the apoptin protein (Xiao et al., 2010). In their studies, the authors showed that the cytotoxic effect of Ad-VT was present in anthracycline resistant breast cancer cell lines and that Ad-VT could restore anthracycline sensitivity by down-regulating MRP1 expression (Li et al.). On the contrary, MDR1 and BCRP levels remain unchanged. Furthermore, since Ad-VT can induce cell death through either apoptosis or autophagy, the authors also explored the second mechanism. They found that MRP1 expression was significantly affected by autophagy inhibition, while it was not modified by apoptosis inhibitors. Therefore, they concluded that Ad-VT restored sensitivity to anthracyclines through autophagy induction. Clearly, the effect was shown to be mediated by mTOR inhibition; When the authors analyzed the expression of the mTOR protein after adding an autophagy inhibitor, they found that inhibition of autophagy significantly increased mTOR activation and that treatment of MCF-7/ADR cells with 60 MOI Ad-VT treatment reversed the effects caused by autophagy inhibitors. This effect was likely mediated by the AMPK pathway and more specifically by the AMPK-mTOR-eIF4F signaling axis. Traditionally, the relationship between autophagy and drug resistance has been divided into two distinct mechanisms and their related effects: one is associated with its protective mechanism against tumor drug resistance, and the other is related to autophagy-induced cell death, which increases tumor sensitivity to apoptosis. In their study, Li et al. could effectively highlight both effects in MCF-7/ADR treated with Ad-VT. It would certainly be interesting to assess whether similar effects can be induced in other types of cancer cells.

Another review article (Lundstrom) on genetically engineered alphavirus vectors, which have been evaluated for prophylactic and therapeutic use for a broad range of cancer indications in various animal models and in several clinical. Although, based on numerous vaccine studies, it has not been possible to demonstrate superiority of any alphavirus system with respect to immune responses or therapeutic efficacy. In most cases, robust immune responses have been obtained, including humoral and cellular responses. Th1-biased immunogenicity confirmed the potential of alphavirus-based cancer vaccine. The possibility to include alphavirus-based delivery of cytotoxic genes, antitumor genes, immunostimulatory genes, apoptosis induced by alphaviruses, and RNA interference in the form of short interfering RNAs and microRNAs expands the possibilities of therapeutic interventions. Moreover, alphavirus vectors can be applied as recombinant viral particles, including replication-deficient, replication-proficient, and oncolytic viruses, as well as RNA replicons and DNA replicons. It has been demonstrated that the stability of RNA and its resistance against degradation can be improved by RNA encapsulation in lipid nanoparticles. Several studies have also confirmed that due to the presence of alphavirus replicons, both RNA replicons and DNA replicons can induce the same immune response at 100 to 1,000 times lower doses compared to synthetic mRNA and conventional DNA plasmids, respectively. Although alphaviruses have shown good safety and efficacy in various animal models, transfer to humans has often generated disappointingly weak immune responses in clinical trials. Several issues such as targeting, delivery, dose optimization, and potential combination therapy need to be addressed.

Newcastle disease virus was one of the novel platforms reviewed by Huang et al., they discussed the biological properties of NDV, the molecular mechanisms of antitumor of oncolytic NDV, and its application in the field of tumor therapy. NDV is among the limited number of viruses that have shown the ability to elicit partial or even complete responses after treatment with a single drug. The enduring nature of these reactions implies that the therapeutic impact of the virus might be based not only on direct oncolysis, but also on its capacity to facilitate long-term immunity. Recent research findings on NDV demonstrate significant potential in both preclinical and clinical trials.

The process of NDV replication takes place inside the cytoplasmic region of the host cell, without integrating into the host genome. This mechanism ensures the preservation of the integrity and safety of the parental virus. The oncolytic nature of Newcastle disease virus (NDV) can be classified as either lytic or nonlytic, as it selectively infects cells that possess a compromised interferon system. This characteristic enhances the safety profile of NDV when utilized as a vaccine. Incorporation of foreign genes is not necessary for NDV to exhibit a potent anticancer impact and maintain persistent expression of foreign genes. The integration of NDV viral therapy with conventional and emerging tumor treatment modalities has been documented and holds significant potential for widespread implementation. However, many inquiries about NDV therapy, similar to other oncolytic viruses (OV), persist without definitive answers. These unsolved questions encompass the practical methodologies for administering NDV therapy, optimal genetic engineering approaches, the therapeutic sequence for immune checkpoint inhibitors, and the most effective combination partners for NDV therapy. At present, there is a lack of established consensus on the most effective and appropriate approach for patients to utilize the virus, both in terms of methodology and timing. The presence of a tumor microenvironmental barrier and the cytoplasmic matrix in solid tumors may limit and suppress virus entry and multiplication, hence reducing its oncolytic efficacy. The presence of an excessive number of foreign genes may have an impact on the replication process of Newcastle Disease Virus (NDV). Furthermore, the NDV purification process requires extensive measures to achieve a clinical-grade virus product. Integrating NDV therapy with conventional and alternative medicines has the potential to emerge as an innovative approach in the field of cancer treatment. The potential increase in the anticancer effect can be achieved by integrating NDV viral therapy with existing immunotherapy, using the immunomodulatory impact of NDV. Consequently, NDV will emerge as a promising candidate for tumor therapy in the near future.

In the next review (Corbett et al.) discussed one such promising oncolytic virus called the Seneca Valley Virus (SVV-001) and its therapeutic implications. SVV development has seen seismic evolution over the past decade and now boasts of being the only OV with a practically applicable biomarker for viral tropism. We discuss relevant preclinical and clinical data involving SVV and how bioselecting for TEM8/ANTXR1, a negative tumor prognosticator, can lead to first of its kind biomarker-driven oncolytic viral cancer therapy. The initial discovery of SVV-001 revealed its specificity for neuroendocrine tumors, highlighting its remarkable capacity to revolutionize the field of neuroendocrine neoplasm therapies. This novel treatment has shown the ability to induce a substantial tumor response, even in cases where immunotherapy was previously believed to be ineffective. However, initial investigations were limited due to the absence of a biomarker that could be used to identify patients who were susceptible to severe viral vasculitis (SVV). The discovery of TEM8/ANTXR1 as a receptor for SVV-001, a therapeutic agent that can be administered through intratumoral injections, in a patient population with a high prevalence of biomarkers, and in conjunction with dual checkpoint blockade to enhance treatment responses, established the foundation for future clinical trials utilizing SVV-001 with a more targeted and strategic approach. The treatment paradigm under consideration was originally designed to address neuroendocrine neoplasms. However, recent advances in understanding the plasticity of neuroendocrine transformation in various solid tumor types, as well as studies revealing widespread upregulation of TEM8/ANTXR1, indicate that SVV-001 may have the capability to target numerous other tumor types that exhibit high resistance to therapy and are associated with high mortality rates. Gaining a greater understanding of the specific immune tumor microenvironment associated with upregulation of TEM8/ANTXR1 in high-grade neuroendocrine carcinoma, well differentiated neuroendocrine tumors, and related tumor types is crucial for effectively using SVV-001 as a therapeutic approach for these conditions. Furthermore, this understanding is essential for the advancement of novel agents that can be used in conjunction with SVV-001.

## 5 Combination therapies

In research work by Obaid et al. employed acarbose (ACA), a specific alpha-glucosidase inhibitor, to induce glucose deficit combined with oncolytic Newcastle disease virus (NDV) to enhance antitumor action. In this study, a murine model of breast cancer was used, in which mammary adenocarcinoma tumor cells (AN3) were subjected to treatment with ACA, NDV, and a combined administration of both compounds. The research includes an investigation of various parameters, including antitumor efficacy, relative body weight, glucose level, hexokinase level (HK-1) determined by enzyme-linked immunosorbent assay (ELISA), glycolysis product (pyruvate), total adenosine triphosphate (ATP), oxidative stress markers (reactive oxygen species and reduced glutathione), and apoptosis assessed by immunohistochemistry. The findings demonstrated an important level of antitumor efficacy after the administration of combination therapy. The observed antitumor activity was associated with a drop in body weight and glucose levels, downregulation of HK-1, inhibition of glycolysis products such as pyruvate and total ATP, activation of oxidative stress characterized by an increase in reactive oxygen species (ROS) and a decrease in reduced glutathione, as well as the appearance of apoptotic cell death. The results suggest a novel approach to combating breast cancer by targeting glycolysis suppression, glucose deprivation, oxidative stress, and apoptosis, with potential therapeutic applications.

In summary, the findings of this investigation provide solid evidence in favor of the innovative hypothesis that ACA triggers glucose restriction, while virotherapy acts synergistically to improve metabolic oxidative stress and induce apoptosis. This study presents novel findings that indicate that ACA-induced glucose restriction acts in synergy with oncolytic NDV, which demonstrates a promising therapeutic approach that targets the glycolysis pathway for enhanced efficacy and safety. The integration of many therapeutic approaches into this unique treatment modality demonstrates a strong potential for application in clinical therapy.

The next article on this Research Topic described that the latest discoveries related to oncolytic adenoviruses (OAds) have the potential to offer a novel approach to improve the outcomes of individuals affected by triple negative breast cancer (TNBC) and other forms of breast cancer (BC) (Green-Tripp et al.). Oncolytic adenoviruses (OAds) have been genetically modified to exhibit the ability to specifically induce lysis, elimination, and activation of host antitumor immune responses, while protecting normal cells from injury. The common modifications observed involve the removal of certain components within the early gene products, such as the E1B55 KDa protein and particular segments of the E1A protein. Alternatively, the introduction of tumor-specific promoters can also be employed as a modification strategy. The efficacy of oncolytic adenoviruses (OAds) in the treatment of several types of adenocarcinomas in patients with breast cancer (BC) has not been adequately evaluated in clinical trials. Preclinical research showed effectiveness in breast cancer cell lines, namely, triple negative breast cancer cells, using innovative adenoviral mutants that showed encouraging results. In this review, Green-Tripp et al. examined the results described for the most promising oncolytic adenoviruses (OAds) in preclinical investigations and clinical trials, both as standalone treatments and in combination with established conventional therapies or emerging therapeutic approaches. The present focus of research involves investigating the efficacy of combining OAds with small molecule medications that target the epidermal growth factor receptor (EGFR), androgen receptor (AR), and DNA damage repair through new PARP inhibitors. These combinations have been shown to exhibit improved efficacy. The co-administration of Olaparib, a PARP inhibitor, with oncolytic adenoviruses (OAds) demonstrated a significant and significant anti-neoplastic response. The most encouraging results have been observed to date when oncolytic adenoviruses (OAds) are used in combination with antibodies targeting immunological checkpoints or expressing cytokines derived from the viral backbone. Although multiple clinical trials and preclinical research have provided evidence of the safety and efficacy of cancer-selective oncolytic adenoviruses (OAds), additional advances are required to effectively eradicate metastatic lesions, enhance immune activation, and promote intratumoral viral dissemination.

In the next review (Shao et al.) concluding the presence of relatively good results of studies in the field of treatment of solid cancers such as gastric cancer using oncolytic viruses, it seems that these viruses can be used more widely in combination therapies to increase the efficiency and effectiveness of cancer treatment. Nevertheless, this therapeutic method has its difficulties and requires more studies. In peritoneal metastasis gastric cancer, virotherapy can limit peritoneal metastasis and tumor metastasis to the peritoneum in diverse ways, such as direct oncolysis of tumor cells, as well as inhibition of mechanisms and molecules involved in angiogenesis. Alternatively, inserting genes with antitumor function into the genome of oncolytic viruses for expression in virus-infected tumor cells can enhance therapeutic effect. Viruses seem to have a wide range of unknown functions, and due to their extraordinary capabilities, such as their ability to replicate in hypoxic conditions, which is one of the drawbacks of cancer therapy, shortly, they can be used to treat cancers to the maximum benefit performance.

## 6 Methods to develop OV quantification

Despite careful quality control, during high titer production, “wild-type-” like replication-competent adenovirus (RCA) contaminants can be generated through recombination events due to the DNA sequence similarity between OV and host cells Gao et al.. These RCA contaminants raise various safety concerns in clinics and detection methods have been developed. Cell culture-based methods have been developed to detect RCA contaminants in replication-deficient adenovirus vectors. These methods were based on the fact that only RCA contaminants, but not the vectors, can grow in and lyse the test cell line. However, these methods are not suitable to distinguish RCA contaminants from oncolytic adenovirus products because both can replicate in test cell lines. The presence of RCA contaminants is then manually judged by microscopic observation, and thus the results may not always be accurate and quantitative. More recently, Gao et al. developed a qPCR-based method to detect and quantify oncolytic adenovirus products. This system takes advantage of the common use of E1B-deleted oncolytic adenoviruses in clinics. Therefore, specific primers have been designed to differentiate between RCA contaminants and E1B-deleted OV. The system turned out to be robust, accurate, and able to detect an extremely small number of RCA contaminants among high-concentration viral particles. In perspective, simply optimizing primers, the use of this tool could be implemented and expanded (Gao et al.).

## 7 In conclusion

It is obvious that oncolytic virotherapy is progressing in steady and wide steps toward being among conventional cancer therapies. Since the approval of more than one type of OV in clinical use and many in clinical trials, more research is expected to focus on increasing the efficacy to be used as a first-line therapy. The development of oncolytic viruses that target specific types of mutations and genetic alterations in cancer cells will help treat difficult tumors clinically and give more hope to cancer patients.

## References

[B1] Al-Shammari A. M. Abo-Altemen R. A. Shawkat M. S. (2021). Cyperus rotundus L. alkaloid extracts enhance oncolytic Newcastle disease virus against digestive system neoplasms. South Afr. J. Bot. 143, 266–273. 10.1016/j.sajb.2021.08.002

[B2] Driever P. H. Rabkin S. D. (2001). Replication-competent viruses for cancer therapy. Basel: Karger Medical and Scientific Publishers.

[B3] Maroun J. Muñoz-Alía M. Ammayappan A. Schulze A. Peng K. W. Russell S. (2017). Designing and building oncolytic viruses. Future virol. 12, 193–213. 10.2217/fvl-2016-0129 29387140 PMC5779534

[B4] Nemunaitis J. Edelman J. (2002). Selectively replicating viral vectors. Cancer Gene Ther. 9, 987–1000. 10.1038/sj.cgt.7700547 12522438

[B5] Russell S. J. Bell J. C. Engeland C. E. McFadden G. (2022). Advances in oncolytic virotherapy. Commun. Med. (Lond) 2, 33. 10.1038/s43856-022-00098-4 35603308 PMC9053215

[B6] Vähä-Koskela M. J. Heikkilä J. E. Hinkkanen A. E. (2007). Oncolytic viruses in cancer therapy. Cancer Lett. 254, 178–216. 10.1016/j.canlet.2007.02.002 17383089 PMC7126325

[B7] Xiao L. Yan L. Zhongmei W. Chang L. Huijun L. Mingyao T. (2010). Potent anti-tumor effects of a dual specific oncolytic adenovirus expressing apoptin *in vitro* and *in vivo* . Mol. cancer 9, 10–12. 10.1186/1476-4598-9-10 20085660 PMC2818692

